# Is treatment for bipolar disorder more effective earlier in illness course? A comprehensive literature review

**DOI:** 10.1186/s40345-016-0060-6

**Published:** 2016-09-09

**Authors:** Katie Joyce, Andrew Thompson, Steven Marwaha

**Affiliations:** 1 Warwick Medical School, University of Warwick, Coventry, CV4 7AL UK; 2 Unit of Mental Health and Wellbeing, Division of Health Sciences, Warwick Medical School, University of Warwick, Coventry, CV4 7AL UK; 3 North Warwickshire Early Intervention in Psychosis Service, Coventry and Warwickshire NHS Partnership Trust, Coventry, UK; 4 Affective Disorders Service (IPU 3-8), Caludon Centre, Coventry, UK

**Keywords:** Bipolar disorder, Early intervention, First episode, Effectiveness, Treatment, Multiple episodes

## Abstract

**Background:**

We aimed to investigate a key element of the early intervention approach; whether treatment at an earlier stage of bipolar disorder is more effective than later in its course.

**Methods:**

A comprehensive literature review using Medline, Embase, Psychinfo, PsycArticle and Web of Science as data sources, with a subsequent narrative synthesis. Study quality was assessed using the Cochrane risk of bias method.

**Results:**

Our search strategy yielded eight primary papers and two meta-analyses (of psychological therapies and Olanzapine) in total representing 8942 patients. Five studies focused on comparisons between first and multiple episode; the others on fewer vs more episode categories. There was a consistent finding suggesting treatment in earlier illness stage resulted in better outcomes in terms of response, relapse rate, time to recurrence, symptomatic recovery, remission, psychosocial functioning and employment. This effect was found for pharmacological (Lithium, Olanzapine, Divalproex) and psychological treatment.

**Limitations:**

There was high risk of selection, performance and attrition bias in most studies. First admission or presentation is unlikely to equate to first episode because of duration of untreated illness. Some patients having experienced multiple episodes could be “treatment resistant”. Study heterogeneity precluded meta-analysis.

**Conclusions:**

Psychological and pharmacological treatment in the early stages of illness is more effective than in later stages of bipolar disorder across multiple domains. There is a first episode and early phase effect. Consistent with the staging model of illness findings provide evidence for the clinical utility of an early intervention approach in bipolar disorder to improve patient outcomes.

## Background

Mental illness causes considerable global disease burden, often affecting patients for their entire lifespan (Whiteford et al. 2013). Bipolar disorder, including subthreshold disorders, affect approximately 2 % of the population (Merikangas et al. 2011). It ranks as a major global cause of disability adjusted life years for 10–24 year olds (Gore et al. 2011), and people with the disorder are seven times more likely to die an unnatural death (Hayes et al. 2015). As well as the significant impact on public health, costs of the disorder are very high (Pari et al. 2014), in part because of the functional losses associated with increasing episode number (Marwaha et al. 2013).

Whilst many expert researchers (Berk et al. 2009; Martin et al. 2013; Salvadore et al. 2008) have suggested early intervention (EI) could reduce the cumulative morbidity, early death and financial costs of bipolar disorder, for this case to be persuasive several pieces of evidence need to align. Failure to make contact with mental health services and receive a prompt correct diagnosis and initiation of treatment appear to be a problem for a range of mental disorders (Wang et al. 2005), but this appears particularly true for bipolar disorder. There are high levels of unmet need in young people with bipolar disorder (Charney et al. 2003). Delays in diagnosis are lengthy (mean 12.5 years), clinically important, and associated with social dysfunction (Matza et al. 2005), as well as an increased risk of lifetime suicidality (Hawton et al. 2005; Nery-Fernandes et al. 2012). Indeed, the delay in diagnosis risks young people receiving inappropriate treatment, which may worsen the condition. Thus, the early diagnosis and early initiation of treatment appear important in bipolar disorder and this would support an EI approach.

If research identifies treatment is most effective earlier in illness course or soon after first episode, then a second and critical evidential strand would support the rationale for EI in bipolar disorder. This would clearly demonstrate that outcomes could be changed by having a focus early in the course of the disorder, thus moulding clinical treatment priorities. Such evidence is likely to support the further development and implementation of EI services for people with bipolar disorder, consistent with the clinical staging approach to managing mental disorders (McGorry 2007).

Therefore, we aimed to review the extant literature, to investigate whether treatment at an earlier stage of bipolar (e.g., after the first episode) is more effective than later in the course of the disorder.

## Methods

A comprehensive review of the literature was completed. An initial scoping review enabled search terms to be selected and refined. This also allowed the development of predetermined inclusion and exclusion criteria for article selection to improve search strategy and to reduce bias in paper selection.

### Sources of information

Using a systematic process, an extensive search of papers catalogued in *Medline, Embase, Psychinfo, Psycarticle,* and *Web of Science* was carried out in October 2015. Papers in the English language were searched, with no criterion for publication date. Following this, all abstracts were downloaded into the *Endnote* referencing system and duplicates were deleted. The inclusion and exclusion criteria were applied to each abstract to create a list of papers for full-text retrieval. The reference lists of the included papers were examined for further papers, and the authors of the most relevant studies were contacted requesting details of any other studies which they thought were important in the area.

### Search terms

The search terms were arranged in groups and included mesh terms, (group 1); bipolar, or mania, or hypomania, or manic depression, AND (group 2); the early treatment, or treatment onset, or the early intervention, or first episode, or multiple episode, or incident, AND (group 3); outcome, or recovery, or relapse, or recurrence, or remission, NOT (group 4); and genetics, or surgery, or dementia, or diabetes, or elderly or cardiovascular disease.

### Inclusion and exclusion criteria

Inclusion criteria were: (a) patients diagnosed with bipolar disorder (any type), (b) the direct comparison of outcome in the same study between treatment received earlier (including at first episode) with later in illness course. We also included studies which had sampled hospital inpatients and compared those earlier vs later in illness course. Exclusion criteria were: (a) non-English language papers, (b) studies in which the only population sampled were children, and (c) case-series data.

### Data extraction

The full text of all included studies was scrutinised and a narrative synthesis completed directed at answering the research aims. Data were extracted on sample size, sampling frame, study design, outcomes, and main results.

## Results

**Fig. 1:**
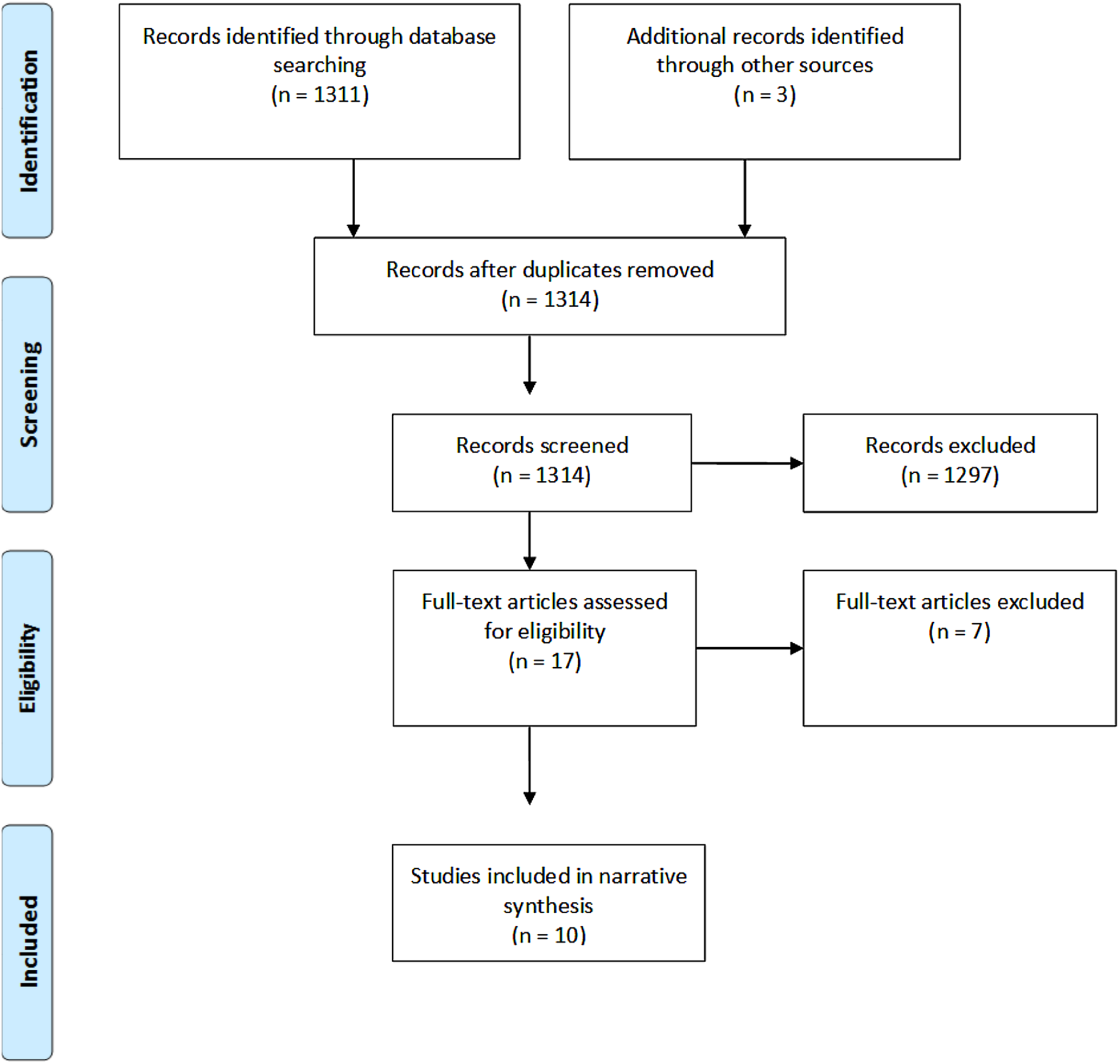
Prisma flow chart

The search generated 1311 abstracts, and after applying the inclusion and exclusion criteria, the full text of 17 papers was retrieved. Seven full-text papers were excluded. The main reasons for study exclusion subsequent to full-text retrieval were either a lack of comparison with earlier or first episode (*N* = 3), or no comparison of treatment between groups (*N* = 2). On contacting relevant authors, one author replied with three study suggestions, one of which was already included and two that did not fulfil inclusion criteria. After reviewing the full-text studies, eight primary studies and two reviews were included for the final review. A PRISMA flowchart (Fig. 1) illustrates the number of studies included and excluded at each stage of the process. Table 1 shows the study characteristics and main findings. The total number of patients in reviewed studies was 8942. We do not present a meta-analytic synthesis of the studies due to heterogeneity and instead structure our findings narratively, reviewing psychological or pharmacological treatment and inpatient studies.

**Table 1:** Characteristics and results of included primary studies and meta-analyses

Author	Sampling	N	Main outcomes measured	Main results
Berk et al. (2011)	Meta-analysis of 12 Lilly funded RCTs	4346	Symptomatic response and relapse using manic symptoms, depression, and global impression	Increased response comparing 1–5 episodes with >10 episode groups in treatment of depression, mania, and maintenance studies
Scott et al. (2007)	Meta-analysis of RCTs	716	Relapse rates	Psychological adjuncts were more effective in patients who had experienced less episodes
Colom et al. (2010)	Patients from the Bipolar Disorders Program at the Hospital Clinic of Barcelona	120	Time to recurrence	Increased response to treatment in people with fewer previous episodes, particularly if less than seven
Dion et al. (1988)	Patients from the clinical evaluation unit of McLean hospital, USA	67	Functioning via the modified vocational status index and modified location code index scales	At follow-up, the first admission had increased rates of employment vs the previous admissions (64 vs 33 %) Admission number not significant in explaining employment in multiple regression
Franchini et al. (1999)	Outpatient Lithium Clinic for Mood Disorders, Milan	270 in total 171 with bipolar disorder	Recurrence rates	Initiating lithium therapy during the first 10 years of bipolar onset results in less recurrence
Jiang (1999)	Acute psychiatric service Taiwan	63	Vocational and residential functioning	The first admission (70.2 %) vs multiple admission (31.8 %) employed and able to live independently at follow-up Effect not significant in multiple regression explaining outcome
Keck et al. (1995)	Patients from the University of Cincinnati Hospital psychiatric units	71	Symptomatic and syndromal outcomes in manic symptoms, depression, global assessment of functioning, and comorbidity	The first episode (using the first admission as proxy) mania associated with shorter hospital stays compared with multiple episode patients
Rosa et al. (2012)	Patients at Santiago Apostol Hospital and in the Bipolar Disorders Program at Hospital Clinic of Barcelona	119	Psychosocial functioning	Treatment of patients in the first episode (using the first admission as proxy) resulted in improved symptomatic and psychosocial outcomes in comparison to patients treated in later episodes, even after controlling for the effects of age and affective symptoms
Tohen et al. (2010)	Inpatients and outpatients initiating or changing oral medication for treatment of acute mania	3115	Recovery, response and remission using clinical global impression, mania symptoms, and depression	Patients treated in their first episode of bipolar reached recovery or remission more often and faster at 12 weeks compared with patients experiencing a later episode
Swann et al. (1999)	Inpatients	154	Response to anti-manic medication	Increased response to treatment for patients who had experienced fewer episodes, particularly if less than ten

**Fig. 2:**
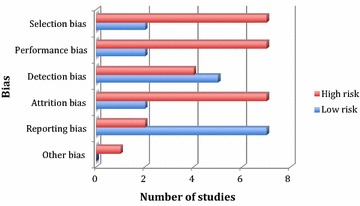
Bar chart to show risk of bias in included studies

Study quality was appraised using the Cochrane risk of bias assessment (Higgins et al. 2008) and an overall view of the quality of the literature can be seen in Fig. 2. There was a high risk of selection, performance, and attrition bias in most studies. However, in the main, studies demonstrated a low risk of reporting bias, providing results for all outcomes that they measured.

### Studies of psychological treatment

Colom et al. (2010) investigated whether treatment response is affected by course of illness progression using the Kaplan–Meier survival analysis. They conducted a post-hoc sub analysis on 120 euthymic bipolar patients, assessing time to recurrence in patients undergoing group psychoeducation in relation to course of their illness. Patients who had experienced six episodes or less demonstrated a reduced time to recurrence (*p* = 0.038) from the addition of psychoeducation to treatment. Patients who had experienced more than seven episodes showed no significant difference in outcome by the addition of psychoeducation. In considering time spent ill, those who had experienced less than six episodes showed a reduction in time spent in any episode polarity with the psychoeducation treatment.

A meta-analysis of the effectiveness of psychological therapies as adjuncts to usual pharmacotherapy was completed by Scott et al. (2007). Data were synthesized from nine studies examining cognitive behaviour therapy, interpersonal social rhythms therapy, family focused therapy, and psychoeducation. Again, they found that adjuncts to treatment were less efficacious in preventing relapse if the patient had experienced more episodes. A significance difference emerged between relapse rates in patients who had experienced twelve episodes or less.

### Studies of pharmacological treatment

Tohen et al. (2010) conducted a two-year prospective observational study, comparing the efficacy of oral medication given after the first episode of mania with oral medication given after multiple episodes. The sample size was large (*N* = 3115). They also investigated the different characteristics of first episode and multiple episode bipolar patients, assessing patients using the Young Mania Rating Scale (YMRS), Clinical Global Impressions-Bipolar Disorder (CGI-BP) mania scores, CGI-BP depression, and the Hamilton Depression Rating Scale. Cox proportional hazard modelling was used as the method of analysis. During the study period, patients treated in their first episode not only reached recovery (anytime during study 37.9 vs 32.0 %, end of 12 weeks 39.6 vs 33.1 %) or remission (first vs multiple episode 89.0 vs 81.4 %) more often, but they did so significantly more rapidly than patients treated in their later episode (*p* = 0.0126). Notably, this was despite patients in their first episode having higher mania ratings (YMRS) than multiple episode patients at baseline. It should be noted that some patients entered into this study were currently medicated (but requiring a change) and the findings refer to episode number as opposed to the duration of illness.

Using a double blind RCT design, Swann et al. (1999) investigated the effect of episode number on response to treatment in 154 inpatients with bipolar disorder. Participants were randomly assigned to Divalproex, Lithium, or placebo medication. To investigate the effect of illness course on response to treatment, the number of the previous episodes was compared using improvement in the manic syndrome score from the Schedule for Affective Disorders and Schizophrenia (SADS) as the main outcome. An improved response to anti-manic medication was evident as measured by the SADS manic syndrome score in patients who had experienced fewer episodes. The pivotal point by which intervention must have been initiated to improve treatment response was approximately ten episodes.

These results are similar to the another investigation of the effect of illness stage on response to lithium prophylaxis. Franchini et al. (1999) sampled a mixed group of patients with mood disorders (*N* = 270), but this included 171 people with bipolar disorder. They found that the earlier commencement of lithium prophylaxis (within the first 10 years of bipolar onset) was associated with improved treatment outcome as measured by recurrence rate of an affective episode even after adjustment for polarity, sex, age, onset, duration of illness, and duration of lithium prophylaxis. Furthermore, the significant association between treatment onset and outcome was stronger for ‘very early’ and ‘early’ lithium onset in comparison to the Lithium initiation after 11 years of preceding illness. The results did not vary between those with recurrent depressive disorder and people with bipolar disorder and polarity of the illness did not mediate the impact of lithium on patient outcome.

A meta-analysis (Berk et al. 2011) reviewed 12 randomised control studies to examine the effect of illness staging on outcome in bipolar disorder with regard to treatment response with Olanzapine. Symptomatic response rates to treatment, as defined by >50 % reduction from baseline, were better in individuals who had experienced less than five episodes with response rate worsening after this point. There was a significant difference in the rates of response for those studies examining mania (52–69 % for <5 episodes vs 29–59 % for >5 episodes). On most outcome measures, the odds of responding were increased by a factor of two in those who had experienced less than five episodes.

The effect was less clear-cut for depression studies, in which rates of response were significantly higher for the 1–5 episode group on only two measures. In terms of studies investigating maintenance treatment, relapse was reduced by 40–60 % for those who had experienced 1–5 episodes or 6–10 episodes compared with the >10 episode group with this being statistically significant with regard to relapse to mania only (*p* = 0.005).

### Studies of hospital inpatient treatment

A number of hospital inpatient studies were identified as providing suitable data. Whilst these studies were not comparing a single treatment between groups, we equated hospital admission with hospital treatment. Whether the treatment was pharmacological and/or psychological was not explicitly stated, but is likely to have involved both.

Sampling patients with bipolar disorder, Keck et al. (1995) report that patients with multiple episodes (*N* = 37) required longer hospital stays to achieve recovery in comparison to people with first episodes (*N* = 34). Furthermore, the increased requirement for treatment was despite similar ratings of illness severity on a variety of measures, including the YMRS, Hamilton Rating Scale for Depression, the Scale for Assessment of Positive Symptoms, and the Global Assessment Scale. The main limitation to the study was one of small sample sizes, though it is noteworthy that the participants were patients with severe symptoms by virtue of the sampling strategy.

Rosa et al. (2012) used a naturalistic design to investigate the differences in psychosocial functioning in 119 hospitalized bipolar patients comparing those experiencing their first episode and multiple episodes. Significantly, more patients in the first-episode group had symptomatic recovery (vs multiple episodes) at 12 months (62.7 vs 44.9 %, *p* = 0.005). Patients being treated in their first episode had better functional outcomes at 6 and 12 months compared with those being treated in later episodes in diverse outcomes, such as autonomy, occupation, cognition, interpersonal relationships, and leisure time even after controlling for the effects of age and affective symptoms at 12 months (all *p* < 0.005).

Dion et al. (1988) investigated the relationship between symptoms and functioning in patients with bipolar disorder (*N* = 67) admitted to hospital at a 6-month follow-up point. Comparing the first admission patients with those with one or more previous admissions, they found that the first admission group had a higher level of independent living at follow-up (85 vs 66 %). In terms of employment, the first admission group also fared better (66 vs 33 %) assessed by the vocational status index, but in a multiple regression analysis, admission number did not predict employment status at 6 months.

Jiang (1999) conducted a prospective study investigating functional outcomes in 63 bipolar disorder patients who were admitted to hospital in Taiwan. In comparison to those who had experienced multiple admissions, the first admission group showed significantly higher vocational functioning (*p* = 0.034) and were more often employed (70 vs 31.8 %). Whilst more of the first admission group were living independently (70 vs 40 %) at year 1, this was not statistically significant (*p* = 0.24). However, the number of admissions was not an independent predictor of vocational functioning after controlling for confounders using a regression analysis.

## Discussion

### Main findings

A comprehensive review of the current literature was completed to investigate whether the effectiveness of treatment of bipolar disorder varies depending on the illness stage. In summary, the literature suggests that treatment earlier in the course of illness is more effective than in the later stages of bipolar disorder. Whilst being based on a small number of studies, this finding is seen for both psychological and pharmacological therapies and the effect is apparent in a range of functional, symptomatic, recurrence, and relapse outcome measures. When confounders were controlled, this effect was attenuated and rendered non-significant in two studies.

### Limitations of the literature

There were several methodological limitations to the literature that frames our findings. We wished to answer the question of whether treatment is more effective earlier in illness course. A suitable methodologically robust study design would be to sample treatment naïve individuals with a first episode and multiple previous episodes of illness and compare treatment effectiveness between the groups. We did not identify any studies using this methodology.

A related point is that many of the identified studies included those with multiple episodes who had already received treatment within the previous episodes. A proportion of these patients may have been “treatment resistant”, defined as having received two consecutive medications without recovery (Gitlin 2006). That group may have by definition, been less likely to respond to treatment in comparison to those with first episode and this could bias results in favour of treatment effectiveness earlier in illness course. In the main, it is unknown what proportion of the populations in these studies could be categorised as treatment resistant. However, in one study, approximately 50 % of the sample had previously found the treatment either ineffective or were intolerant to it (Bowden et al. 1994; Swann et al. 1999).

Studies from which relevant data could be extracted showed substantial variations in study design, sampling frames, analytic strategies, and outcomes measured. In addition, whilst nine studies used episode number as the category for comparison, one study reported length of illness (e.g., less than 10 years). Because of this heterogeneity in the literature, we did not statistically combine results in a meta-analysis, as in our judgement, this not feasible or considered potentially meaningful. Instead, we completed a narrative synthesis as the most methodologically sound way to understand the underlying patterns in the literature given the limitations described above. This type of synthesis enabled a richer understanding of the extant literature.

There may have been clinical and socio-demographic differences between the first and multiple episode groups, which could have had independent effects on outcome (Berk et al. 2011; Tohen et al. 2010) whether these were measured or not. Confounders, such as age, have been controlled for in some analyses and significant differences remain between the groups (Franchini et al. 1999; Rosa et al. 2012). In other studies, despite striking absolute differences in outcome (e.g., employment) between the first and multiple admission groups, admission number did not remain significant after controlling for other factors (Jiang 1999). Variable adjustment for confounding and disparate analytic strategies (due to the nature of studies included) means that caution is needed in direct comparisons between studies.

How first episode was classified frequently relied on a first admission to hospital with four studies using the first admission as a proxy for first episode. This is important given that prior to clinical diagnosis, patients have often experienced the previous affective symptoms (Martin et al. 2013) and duration of untreated illness can be lengthy (Berk et al. 2007; Murru et al. 2015). This is the case even after presentation to mental health services (Patel et al. 2015). There is a danger then in reviewing the literature that first episode status is conflated with the first admission or first contact with medical services, when, in fact, this is not the case. This potential variation between studies means that caution should be exercised in conclusions, regarding episode number and treatment effectiveness. It is also difficult to make direct comparison between the bulks of studies that investigate the impact of episode number on treatment effectiveness with a study that details length of illness (Franchini et al. 1999) as the variable analysed.

There were only five studies that compared people with first episode bipolar (with four relying on the first admission for this categorisation) and those with further episodes. Therefore, the current available literature (which included two meta-analyses) is weighted towards the comparisons of people who have already experienced a number of episodes with those having experienced more.

Finally, there may be a treatment confounding effect apparent in our results. For example, patients with the first episode bipolar disorder or in the early stages may have received more robust care than patients in comparison groups given the high priority now given globally to first episode mental disorders. This, of course, is less likely to be an issue for older studies.

### Effectiveness of treatments in multiple domains

Psychological or pharmacological treatments at an earlier stage of bipolar disorder are more effective that in the later stages. This is apparent in multiple domains covering outcomes of importance to both clinicians and patients. The literature spans greater effectiveness on relapse, remission, recovery rates, comorbidity, symptomatic and syndromal outcome, global psychosocial functioning, and vocational and residential functioning. The fact that the same trend is seen with different treatment modalities, as well as a variety of outcomes adds validity and potency to our findings.

### Timing

The evidence base in the first episode psychosis suggests that using a stage-specific approach to treatment in first episode of illness is more effective than not (Marshall et al. 2011). The underlying tenet of this approach was supported by the current literature review. However, a number of studies, including the two meta-analyses, suggest even after a first episode, less episode number is associated with greater treatment effectiveness. This finding was independent of treatment studied (Lithium, Olanzapine, Divalproex, CBT, psychoeducation) or study design (Berk et al. 2011; Colom et al. 2010; Scott et al. 2007; Swann et al. 1999).

The pivotal point at which earlier treatment was more effective ranged from 5 to 12 episodes. Given the differences between studies and the limitations of the literature, it is difficult to be sure that a pivotal episode number exists between this range after which effectiveness changes or whether this is simply a function of how data were categorised and analysed. Further research is necessary to definitively answer this question.

The early treatment would seem important, but our findings suggest an early phase effect as opposed to a solely first episode effect. A possible interpretation is that for some people, it takes a number of episodes to achieve medication optimisation and adequate adherence and to be able to engage fully in therapy. Whilst the early intervention services for bipolar disorder and their evaluation are in their infancy (Marwaha et al. 2016), evidence from an RCT sampling people early in illness course does suggest that specialised and systematic treatment is more clinically and cost-effective than the standard outpatient care (Kessing et al. 2013). The findings of this review would support an extension of this approach.

### Why does treatment earlier in illness course improve outcomes?

The clinical staging approach for bipolar disorder suggests a model, in which there is a progression from “at risk” symptoms to the first presentation, to multiple episodes right through to refractory illness (Kapczinski et al. 2009). Movement through the stages can be due to a combination of genetic vulnerability, life stresses, and substance misuse, and each stage may be linked to abnormalities in biomarkers, such as TNF-alpha, BDNF, and 3-nytrotyrosine (Kauer-Sant’Anna et al. 2009). Advancing illness stage is associated with neuroprogression evidenced by the changes in the brain structure (especially in the fronto-limbic system) (Berk et al. 2011; Mwangi et al. 2016). Alongside these biological changes, there is evidence for a progressively smaller inter-episode period, as episode number increases. Whilst the earlier episodes may need to be triggered, the illness progresses episodes can begin to emerge spontaneously. This has been conceptualized into a stress sensitization-kindling model of bipolar disorder, in which repeated abnormal brain activity reduces the threshold for repeat events increasing the risk of relapse (Post 2007).

These factors are very likely to form part of the explanation for our findings that treatment early in illness course is more effective than in later episodes in terms of both clinical and symptomatic outcomes. This review suggests that the progression to later stages of illness is associated with treatments becoming less effective and these chimes with the requirement for more complex treatment regimes for many people who have well-established bipolar disorder (Post et al. 2010). The greater effectiveness in improving functional outcomes in the early course may be particularly linked to the initial appearance and worsening of cognitive impairments with time, a factor which is known to independently predict vocational functioning in bipolar disorder (Gilbert et al. 2013; Torres et al. 2011). Our findings also paradoxically highlight the scale of the therapeutic challenge to assist people in later stages of the illness, in which there appears to be some level of treatment resistance.

To conclude, this literature review found substantial evidence that both pharmacological and psychological treatments for bipolar disorder are more effective in the earlier stages of illness. The effect, which is demonstrable at the first episode, is also apparent in the early phases of treatment. The findings provide some evidence for the clinical and policy rationale of an early intervention approach in bipolar disorder to improve patient outcomes.

## Abbreviations

EI: early intervention
YMRS: Young Mania Rating Scale
CGI-BP: Clinical Global Impressions-Bipolar Disorder
SADS: Schedule for Affective Disorders and Schizophrenia
